# Socioeconomic and ecological drivers of contagious pig diseases in free-range systems: Evidence from Gwembe Valley, Zambia

**DOI:** 10.1371/journal.pntd.0014523

**Published:** 2026-07-09

**Authors:** Catherine Besnart Dzombe, Herman Chambaro, Karl Rich, Magda Rich, Chisoni Mumba

**Affiliations:** 1 Department of Disease Control, School of Veterinary Medicine, University of Zambia, Lusaka, Zambia; 2 Department of Pathobiology, College of Veterinary Medicine, University of Illinois Urbana–Champaign, Champaign, Illinois, United States of America; 3 Department of Agricultural and Applied Economics, College of Agriculture and Life Sciences, Virginia Tech, Blacksburg, Virginia, United States of America; 4 College of Veterinary Medicine, Oklahoma State University, Stillwater, Oklahoma, United States of America; University of Calgary, CANADA

## Abstract

Free-range pig systems support rural livelihoods in Zambia but increase exposure to contagious diseases through unrestricted movement, environmental contamination, and informal trade. We explored non-biological drivers of disease risk in Gwembe District to inform practical control strategies. We used Spatial Group Model Building (SGMB) with LayerStack mapping to engage 12 purposively selected value-chain actors (farmers, traders, transporters, and veterinary personnel). Participants co-produced spatial layers (settlements, production zones, markets, disease occurrence, service points), behavior-over-time graphs, and causal loop diagrams (CLD). Findings highlighted intersecting non-biological drivers: free-range husbandry, open defecation and shallow-well contamination, seasonal water scarcity, limited veterinary access, informal marketing, and economic pressures that shape herd management and pig movement. Discrepancies between transporters’ reported volumes and district records suggested unreported flows that could undermine surveillance. Participants recognized risks consistent with African swine fever (ASF) spread via pig movement and porcine cysticercosis (PCC) via sanitation failures, but awareness of PCC pathways was limited. Disease dynamics in free-range systems arise from feedback across human practices, animal behavior, environment conditions, within markets interactions acting as key transmission paths that shape both local persistence and wider disease spread. Low-cost housing, community sanitation, participatory surveillance, and tailored farmer education are feasible entry points for improving disease prevention and control in free-range pig production systems. In Gwembe valley, SGMB effectively elicited local knowledge and revealed leverage points for integrated One Health interventions that align with community realities and can strengthen rural livelihoods. Disease dynamics in free-range systems arise from feedback across human practices, animal behavior, and environmental conditions, with market interactions acting as key transmission nodes that shape both local persistence and wider disease spread.

## Author summary

Free-range pig farming is common in rural Zambia and helps many families earn income and improve food security. However, when pigs roam freely, they can easily come into contact with waste, other animals, and informal markets, which increases the risk of disease spread. Two important diseases in such systems are African swine fever, which kills pigs and affects farmers’ livelihoods, and porcine cysticercosis, which can also infect people and is linked to poor sanitation. We worked with farmers, traders, transporters, and veterinary staff in Gwembe District to understand how everyday practices, environment, and trade patterns influence disease risks. Instead of focusing only on biology, we looked at how non-biological realities shape pig management. Participants showed that poverty, limited veterinary services, seasonal water shortages, and informal pig trade all contribute to disease risk. They also highlighted gaps in disease awareness and reporting. Our findings suggest that improving pig health is not only about medicines. Better sanitation, simple pig housing, farmer education, and community-based surveillance can reduce disease risks while fitting local livelihoods. This study shows that locally informed, participatory approaches can support healthier pigs, safer food, and more resilient rural communities.

## 1. Introduction

Pig farming is increasingly recognized as a viable avenue for income generation, food security, and employment creation worldwide [1]. This growth is driven by several advantages over other livestock species, including high reproductive efficiency, short generation intervals, adaptability to diverse environments, and high feed-to-meat conversion ratios [1].

In Zambia, pig production is mainly practiced under three rearing systems: free-range (extensive), semi-confined (semi-intensive), and confined (intensive) [2]. In the free-range system, pigs scavenge for food with little to no supervision or housing. Semi-confined systems involve partial shelter, with pigs allowed to roam within a restricted area. In contrast, the confined system keeps pigs fully housed indoors, with controlled feeding and management [2]. Pig production in Zambia is projected to reach approximately 279,820 heads by 2026, with Eastern and Southern provinces contributing most of this growth [2,3]. Despite this growth, over 80% of pig farmers rely on traditional free-range systems, which are characterized by low productivity [4,5]. These system predominantly involves indigenous breeds such as Lusitu and Nsenga, which constitute 65% of the national pig population alongside exotic breeds [6]. Although less productive, indigenous breeds are preferred for their low production costs and resilience to harsh environmental conditions, including variable weather and limited feed resources [6,7].

Despite these advantages, free-range pig production is highly vulnerable to disease outbreaks, which can significantly disrupt growth and profitability [8]. Housing and management conditions, including biosecurity, hygiene, ventilation, and temperature control, directly influence pigs’ exposure to pathogens and their immune resilience, especially for diseases such as African swine fever (ASF) and porcine cysticercosis (PCC) [8–10].

ASF is a highly contagious, hemorrhagic disease of domestic and wild swine caused by the African swine fever virus (ASFV), the only member of the family *Asfarviridae*, genus *Asfivirus* [11]. In sub-Saharan Africa, ASFV is maintained in a sylvatic cycle involving warthogs and soft ticks (*Ornithodoros moubata*) complex [2]. However, domestic pig-to-pig transmission occurs where the sylvatic cycle is absent [2]. ASF is considered the most significant global threat to pig production, with mortality rates approaching 100% [12]. While it poses no direct risk to human health, ASF has severe socio-economic impacts, including loss of income due to pig mortality and culling, food insecurity, and market disruptions [13,14]. These effects particularly more detrimental on traditional pig farmers [12,13].

PCC is caused by *Taenia solium*, a zoonotic parasite responsible for taeniasis and cysticercosis in humans and pigs [15]. While PCC is an important animal health and livestock productivity concern, it is also a major public health issue because it forms part of the transmission cycle of *Taenia solium*. Human taeniasis occurs through the consumption of undercooked infected pork, whereas ingestion of *T. solium* eggs, often facilitated by poor sanitation and hygiene, can lead to cysticercosis in both humans and pigs [15,16]. In humans, cysticercosis may progress to neurocysticercosis, one of the leading preventable causes of epilepsy in endemic regions, disproportionately affecting resource-constrained rural populations with inadequate sanitation infrastructure [17].

Consequently, PCC represents a NTD situated at the intersection of livestock production systems, sanitation, poverty and human health inequities [17]. Recognizing its public health importance, Zambia has identified it as one of the seven priority NTDs, with a prevalence of 15.2% in Southern Province [18,19].

Gwembe Valley’s predominance of free-range pig systems, poor sanitation, limited veterinary services, and seasonal water scarcity create conditions that sustain and amplify transmission of both ASF and PCC [18,20]. For this reason, this study aimed to investigate the non-biological determinants that influence the occurrence and spread of contagious disease of pigs in free-range pig value chains in Gwembe District of the Southern Province of Zambia. In this study, non-biological determinants refer to the socio economic (poverty, income stability), environmental (water scarcity, sanitation), behavioural (free range husbandry, open defecation), institutional (limited veterinary access), and trade-related (informal pig movement) factors that indirectly shape disease transmission dynamics beyond the pathogen-host interaction itself. This aim was achieved through the following specific objectives: firstly, mapping pig value chain actors in Gwembe District. Secondly, understanding pig production and marketing practices among value chain actors, and thirdly, investigating the non-biological determinants of ASF and Porcine cysticercosis using Spatial Group Model Building (SGMB) facilitated by the LayerStack.

While livestock disease management traditionally focuses on biological factors, which targets the pathogen, host and direct biological mechanism of transmission, the importance of non-biological determinants is increasingly recognized [21]. The Gwembe Valley, characterized by its semi-arid ecological conditions, seasonal water scarcity, and a predominantly subsistence-based socio-economic context marked by traditional free-range pig production [18,22], provides a valuable case study. Here, factors such as climate, market dynamics, agricultural practices, cultural norms, and household socio-economic status intersect to shape disease epidemiology [23].

Systems thinking is a problem-solving approach that analyses complex issues by examining the interactions among interconnected elements within a system. A system is a collection of elements working together toward a shared objective. This approach acknowledges that a system’s behaviour is frequently emergent, surpassing the cumulative impact of its individual components, highlighting the significance of considering the system holistically [24]. Using a systems thinking approach, this study applies SGMB facilitated by LayerStack an innovative tool to explore these dynamics. The findings aim to inform integrated, community-centered interventions that address both biological and non-biological disease determinants, thereby improving the health and productivity of free-range pig populations in Gwembe and similar contexts worldwide.

## 2. Materials and methods

We obtained ethical approval from Excellence in Research Ethics in Science (ERES) Converge (Ref. 2023-May-009). Because some participants preferred anonymity and had varying literacy levels, we sought verbal informed consent, as approved by the ERES Converge ethics committee. The facilitator documented consent from all farmers, traders, transporters, and veterinary personnel. We did not collect any personally identifying photographs or audio recordings. We anonymized all maps and notes and stored them on password-protected devices accessible only to the research team.

### 2.1. Study design

This research employed a qualitative case study design to examine the problem through a systems thinking approach. The case was defined as the free-range pig production and marketing system in Gwembe District of Zambia, viewed as a socio-ecological and value-chain system through which multiple actors interact and influence the occurrence and transmission of contagious pig diseases. This case was bounded geographically (Gwembe District), temporally (October 2023), and by the stakeholders and practices associated with pig husbandry, trade, and disease management in the district. To achieve the study objectives, data were collected through document review, key informant interviews (KIIs), and Spatial Group Model Building (SGMB) facilitated through LayerStack. The KII involved engaging a purposively selected individuals who were likely to provide relevant information and insights on the subject matter, guided by an interview guide as described by Kumar [25]. The document review entailed analysing official records and reports, such as monthly reports on livestock production, population, movement, animal health and trade in the district, obtained from the District Veterinary Office (DVO).

### 2.2. Description of study sites

The research was conducted in Gwembe District, situated approximately 93.5 km east of Choma, the capital of Zambia’s Southern Province. Gwembe is bordered by Siavonga District to the east, Monze to the north, Pemba to the west, and Lake Kariba to the South. The district features a complex topography comprising a valley and a plateau, with the northern region characterized by flatland interspersed with hilly formations and significantly eroded limestone outcrops. The predominant rocky landscape limits the availability of arable land suitable for extensive agriculture [4].

According to the 2022 national census, Gwembe’s population stands at approximately 79,273 individuals, resulting in a population density of 19.9 persons per km^2^ [26]. The district’s rugged terrain and scarce pasture resources render it unsuitable for large ruminant farming, contributing to the lowest cattle population within the province. Nevertheless, Gwembe supports substantial populations of small livestock, particularly pigs and goats, with a documented pig population of 9,188 as of 2019 [4].

The demographics of Gwembe predominantly comprise rural and peri-urban settlements, where a significant portion of households experience poverty and lack access to essential sanitation and potable water [4]. The local economy is primarily driven by trade and subsistence agriculture. Key food crops cultivated in the area include millet, sorghum, and maize, with groundnuts being intercropped alongside various vegetables such as cucumbers, pumpkins, tomatoes, melons, rape, and cowpeas. Cotton serves as the principal cash crop for the district [27]. Recently, the fishing sector has emerged as an increasingly vital economic activity, particularly following the deployment of fishing cages in Lake Kariba by the Ministry of Livestock.

**Fig 1:**
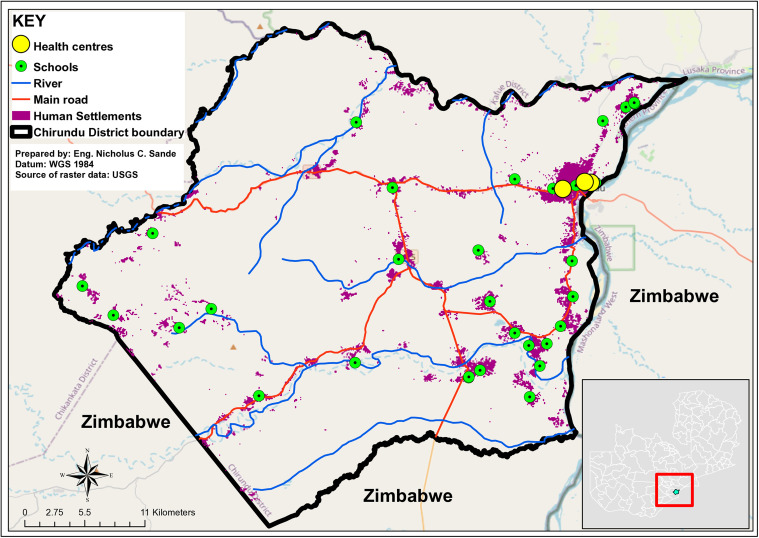
Study area in Gwembe District, Zambia. Map showing the distribution of veterinary camps. The map was developed by the authors using ArcGIS version 10.3 (ESRI, Redlands, CA, USA), with basemap data from OpenStreetMap (OpenStreetMap contributors) and satellite imagery sourced from the USGS EarthExplorer platform (U.S. Geological Survey; https://earthexplorer.usgs.gov/.

In terms of livestock, the district in manned by one veterinary officer and eight veterinary assistants in eight camps as shown in Fig 1. A veterinary camp is a function unit of livestock production in Zambia as described by Mumba et al. [28].

### 2.3. Sampling and selection of participants

The stakeholder meeting consisted of 12 purposively selected participants (Table 1), guided by the approach described by Mumba et al. [28]. Purposive sampling was used to recruit information-rich participants occupying critical positions within the pig value chain. In qualitative systems research, emphasis is placed on depth of contextual insight and diversity of system perspectives rather than statistical representativeness [29]. Participants were individuals actively engaged in the Gwembe pig value chain. The group comprised four pig farmers, each representing one of the four major production zones in the district (East, West, North, and South), five pig traders (four from high-production areas and one from the district headquarters), two principal transporters involved in live-pig movement, and the district’s sole veterinary assistant.

**Table 1:** Stakeholders in the Gwembe pig value chain.

Role in Pig Value Chain	Region	Education level	Sex	Age	Duration of stay in Gwembe	Duration of pig business
Trader/Farmer	Gwembe Central	Junior Secondary	Female	46	26	15
Farmer	Gwembe Central	Tertiary	Female	40	6	4
Trader/Farmer	Bboondo	Primary	Female	41	41	4
Farmer	Gwembe Central	Secondary	Male	63	28	4
Transporter	Chipepo	Junior secondary	Male	39	39	20
Trader/Farmer	Munyumbwe	Primary	Male	40	40	15
Farmer	Munyumbwe	Secondary	Male	42	7	5
Trader/Farmer	Gwembe Central	Junior Secondary	Female	50	50	15
Transporter/Farmer	Bboondo	Secondary	Male	29	29	8
Trader/Farmer	Munyumbwe	Junior Secondary	Male	56	46	15
Farmer	Bboondo	Secondary	Male	44	44	10
Veterinary Assistant	Gwembe Central	Tertiary	Male	50	43	23

Note: The district has no formal pig processing plant, however, the traders informally slaughter pigs in their backyards. Gwembe district only has traditional pig farmers.

Participant identification and recruitment were facilitated by local gatekeepers. Gatekeepers represent the link between the facilitators of spatial group model building meetings and stakeholders and thus play a brokering role and control access to the community [30,31]. The gatekeepers were veterinary assistants and community leaders who recruited the 12 participants [32]. The researchers (authors) did not select specific individuals; instead, they provided eligibility criteria and requested representation across key value-chain roles: farmers, traders, transporters, and veterinary personnel. Gatekeepers then distributed invitations and oriented potential participants on the study purpose during meetings. This approach ensured that those selected were directly involved in the free-range pig system and possessed relevant contextual knowledge, while preserving community-driven participant selection.

### 2.4. Data collection

We sourced epidemiological data concerning ASF and PCC prevalence, distribution, control measures, and livestock movements from the District Veterinary Office through a comprehensive document review and KII. Subsequently, we employed the SGMB framework, utilizing the LayerStack facilitation tool as described by Mumba et al. [28] and Lie & Rich [33], to investigate the dynamics of contagious pig diseases within the district.

At the core of the discussion, the LayerStack was positioned on the table, encased within a transparent sleeve alongside the regional study map. A total of five acetate layers (modules) were systematically applied, representing: (i) human settlements, (ii) pig production areas, (iii) pig/pork market locations, (iv) pig diseases (ASF and PCC) occurrence rates, and (v) agrovet store distributions. Stakeholders and processes involved in the pig value chain were illustrated using color-coded stickers in various shapes.

Participants developed behavior-over-time (BOT) graphs at each facilitation stage to discern trends. These visual tools were instrumental in revealing potential causal relationships by depicting the temporal dynamics of variables rather than merely highlighting isolated occurrences. We used a semi-structured Focus Group Discussion (FGD) guide throughout. The SGMB session reached thematic sufficiency when no substantially new system drivers or relationships emerged during mapping and discussion. Table 2 shows the agenda for the SGMB session.

**Table 2:** Agenda for the SGMB session.

Time	Public schedule	Detailed scheduled
08:00 - 08:50	Arrival of stakeholders	Scene preparation
09:00 - 09:30	Introductions	-Who are the facilitators? -Where are they coming from? -Why are they in Gwembe district? -Why the stakeholders have been summoned? -Concept of focus group discussions -Concept of layer stack model -House rules
09:30 - 12:00	Layer stack definition and FGD process	-Presentation and definition of each acetate theme. -Each stakeholder to identify their position on the map. -Stakeholders to discuss how settlements have changed over time and what they assume could be the influencing that change. -Stakeholders to identify and discuss areas where pig production, trade, support and disease are mostly

### 2.5. Data analysis

We analyzed the data using an integrated approach that combined SGMB with thematic analysis of insights generated at each step of the LayerStack process [34]. Through SGMB, we iteratively collected, interpreted, and synthesized data by engaging stakeholders in structured participatory modeling sessions. As participants shared their experiences and perspectives, we refined system components and relationships in real time, enabling collaborative sense-making.

We translated information from each LayerStack layer into emerging themes and sub-themes, which were identified through an iterative, inductive process. Information from each LayerStack layer was reviewed to identify recurring patterns and issues, which were grouped into preliminary themes using participants language. Broader themes were then disaggregated into sub-themes to capture specific drivers and variations [34]. All themes and sub-themes were validated through participant dialogue and consensus. We then synthesized these qualitative insights into causal loop diagrams to illustrate feedback relationships and systemic drivers. Our analysis focused on identifying non-biological determinants influencing contagious pig diseases, particularly ASF and PCC within the free-range pig production system in Gwembe District. The SGMB modules represented the overarching thematic domains, while stakeholder-generated insights formed the sub-themes.

### 2.6. Theoretical framework

This study was informed by a constructivist and interpretivist paradigm is grounded in the assumption that reality is socially constructed through human interaction and interpretation, rather than existing as a single objective truth [29]. This was complemented by critical realism and systems thinking [35] within a One Health lens [36]. The constructivist–interpretivist foundation guided the study design and analysis by emphasizing participant perspectives, meanings, and contextual understanding of pig disease risks. Data analysis focused on the co-construction of knowledge, privileging lived experiences [37] and tacit knowledge of farmers, transporters, traders, and district animal health personnel.

A critical realist lens informed the layered analytical approach, where emergent themes and causal loop diagrams were interpreted not only at the level of observed practices but also linked to deeper generative mechanisms, such as structural resource constraints, sanitation systems, informal market governance, and environmental exposure pathways.

**Fig 2:**
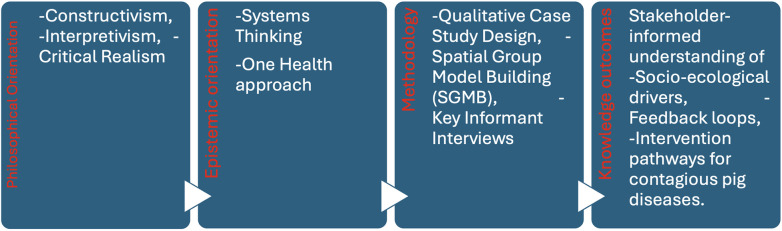
Schematic diagram of the Conceptual Framework (developed by authors).

Systems thinking and One Health principles shaped the mapping process, enabling identification of reinforcing and balancing feedback loops across animal health behaviors and non-biological dynamics. The combination of these philosophical positions allowed the study to move beyond description toward explanation, highlighting how structures, agency, and context interact to shape contagious pig disease risks in free-range systems. The conceptual flow is illustrated in Fig 2.

### 2.7. Data triangulation and integration

Data triangulation was conducted by comparing and integrating findings across document review, KIIs, SGMB discussions, LayerStack outputs, and behaviour-over-time graphs as shown in Table 3. Institutional records from the District Veterinary Office were used to contextualize and validate stakeholder narratives regarding pig movement, disease occurrence, and veterinary access. Spatial patterns identified during LayerStack mapping were cross-checked against participant discussions and official records. Areas of convergence strengthened interpretation of system drivers, while discrepancies, such as differences between reported and officially recorded pig transport volumes, were treated as analytically important findings that highlighted potential surveillance and reporting gaps. This triangulated approach enhanced the credibility, dependability, and contextual validity of the findings by integrating multiple perspectives and forms of evidence within the same socio-ecological system

**Table 3:** Data triangulation.

Data Source	Purpose	Contribution to Analysis
Document Reviews	Review livestock movement and disease reports	Provided institutional and epidemiological context
KII	Explore veterinary and governance perspective	Identified surveillance and service delivery constraints
SGMB sessions	Elicit stakeholder system understanding	Identified socio-ecological drivers and feedback relationships
LayerStack maps	Spatial visualization	Validated spatial distribution of production, trade and disease
BOT graphs	Temporal understanding	Revealed seasonal and behavioural trends
CLDs	Systems synthesis	Integrated drivers into feedback structures

## 3. Results

We synthesized insights from the SGMB sessions and thematic analysis to show how non-biological factors interact to shape contagious pig disease dynamics in free-range systems. Instead of examining individual drivers in isolation, we worked with participants to identify interconnected processes and feedback loops influencing pig movement, husbandry practices, sanitation, and disease exposure. We organized the findings around the core thematic domains that we co-developed with community actors and illustrated them with causal loop diagrams that depict the systemic pathways driving disease risks.

### 3.1. Spatial organization of pig value chain

**Fig 3:**
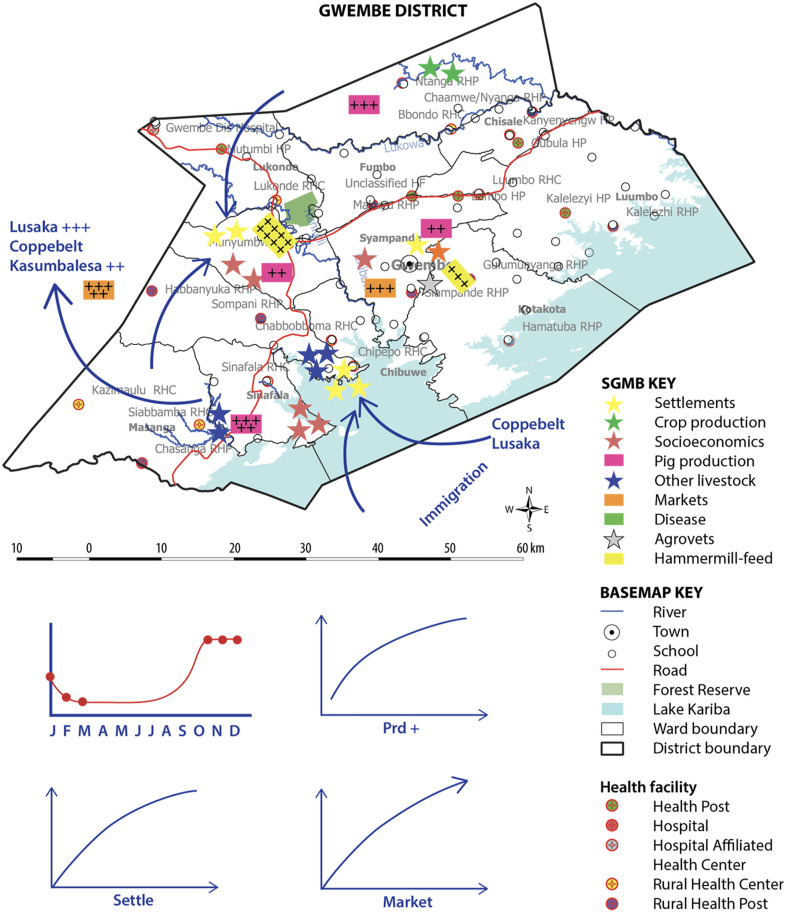
Spatial distribution of settlements, pig production areas, and key value chain features in Gwembe District. The map was developed by the authors using ArcGIS version 10.3 (ESRI, Redlands, CA, USA), with basemap data from OpenStreetMap (OpenStreetMap contributors) and satellite imagery sourced from the USGS EarthExplorer platform (U.S. Geological Survey; https://earthexplorer.usgs.gov/.

The SGMB process identified marked spatial variation in settlement patterns, pig production, and market activities across Gwembe District (Fig 3). Stakeholders identified Chipepo, located along Lake Kariba, as the most densely populated area and an important economic hub associated with fishing activities and migration from neighboring districts and Zimbabwe.

Munyumbwe and Gwembe Central were identified as secondary population centers. Participants reported that livestock keeping, fishing, and crop production influenced settlement patterns across the district. Areas characterized by higher household income levels were primarily located in Chipepo and Munyumbwe, whereas Gwembe Central was associated with formal employment opportunities.

The spatial mapping exercise further showed that pig production, markets, veterinary services, and disease occurrence were unevenly distributed across the district, creating distinct patterns of interaction among value-chain actors.

### 3.2. Pig production and husbandry practices

Participants consistently reported that free-range husbandry was the dominant pig production system throughout Gwembe District. According to stakeholder estimates and DVO records, Sinafala had the highest pig population, followed by Bbondo, Munyumbwe, and Gwembe Central.

Stakeholders reported that free-range management was preferred because pigs could obtain feed through scavenging and access natural cooling areas during periods of high temperature. Most farmers kept indigenous pig breeds, particularly the Lusitu and Gwembe, while a smaller number raised exotic breeds such as Large White pigs.

Open defecation was frequently discussed during SGMB sessions. Participants reported that pigs commonly accessed areas contaminated with human waste. Stakeholders also described seasonal movement of surface runoff from upland areas to lower agricultural zones and shallow wells.

No formal pig slaughter facility was identified within the district. Participants reported that slaughtering typically occurred informally at household level.

### 3.3. Trade and market dynamics

Stakeholders reported that most pigs produced in Gwembe were sold outside the district, with Lusaka and Kasumbalesa identified as the principal destinations.

Transporters indicated that they moved approximately 150–200 pigs per trip to Lusaka and 250–350 pigs per trip to Kasumbalesa, often making multiple trips per week. Participants reported that pig sales increased between August and February before declining during the rainy season.

Document review revealed notable differences between stakeholder-reported pig movements and official veterinary records. District records documented substantially lower numbers of pigs transported during the same period and contained no records of pig shipments to Lusaka during the first two quarters of the year.

### 3.4. Disease awareness and transmission pathways

Participants reported limited direct experience with ASF outbreaks within their own herds. Knowledge of ASF was primarily derived from outbreaks reported in neighboring districts through media sources and veterinary communication channels.

Awareness of PCC transmission pathways was generally low. While participants recognized pigs as scavenging animals that frequently consumed human waste, few participants demonstrated detailed understanding of the role of Taenia solium in transmitting cysticercosis between humans and pigs.

Stakeholders identified several potential disease transmission pathways, including: free-range pig movement, informal trade networks, open defecation, environmental contamination, limited veterinary access, and seasonal water scarcity. These pathways were subsequently incorporated into the SGMB model-building process.

### 3.5. System drivers identified through SGMB

The SGMB process identified five interconnected system drivers influencing disease transmission within the pig value chain: Household socio-economic status, free-range husbandry practices, sanitation and environmental contamination, informal pig trade and movement, and access to veterinary services and disease information. Stakeholders consistently described these drivers as interacting rather than operating independently. For instance, household income influenced the ability to implement confinement systems, access veterinary services, and invest in sanitation infrastructure.

### 3.6. Feedback structures and leverage points

**Fig 4:**
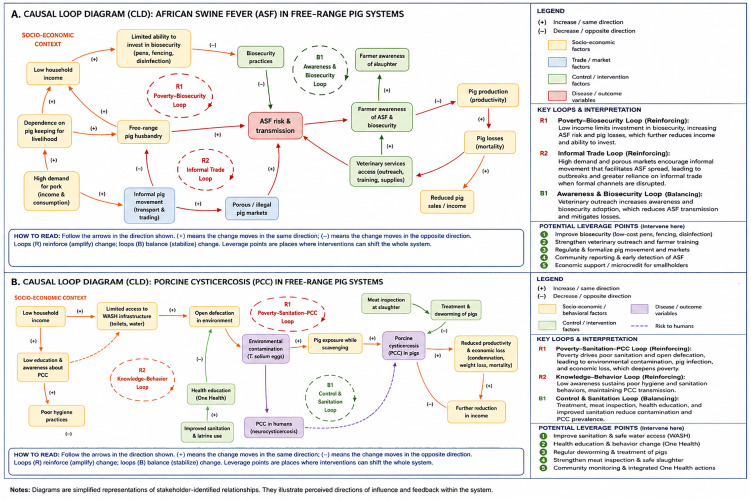
A and B: Causal loop diagrams of ASF and PCC generated from LayerStack through SGMB.

The PCC CLD identified two dominant reinforcing feedback structures as shown in Fig 4b. The first linked low household income, inadequate sanitation, open defecation, environmental contamination, and PCC occurrence. Stakeholders described how limited resources constrained access to improved sanitation facilities and veterinary services. The second reinforcing structure linked low disease awareness with continued risky sanitation and husbandry practices. Participants also identified balancing processes involving treatment, vaccination, and improved sanitation practices that could potentially reduce disease transmission.

#### 3.6.1. Feedback structures associated with ASF.

The ASF CLD highlighted interactions between income, biosecurity, free-range husbandry, pig movement, and market activities as shown in Fig 4A.

One reinforcing structure linked low household income to reduced investment in biosecurity, increased disease exposure, and subsequent economic losses.

A second reinforcing structure linked informal trade networks and unregulated pig movements with potential disease spread beyond the district.

Stakeholders identified veterinary outreach, surveillance, and farmer education as potential intervention points within the system.

## 4. Discussion

This study used SGMB facilitated through LayerStack to examine how socio-economic, environmental, behavioural, and institutional factors interact to shape the transmission dynamics of ASF and PCC within free-range pig systems in Gwembe District, Zambia. Rather than examining disease occurrence through isolated biological pathways alone, the study explored how disease risks emerge through interconnected non-biological systems involving livelihoods, sanitation, trade, environmental conditions, veterinary access, and local knowledge systems. The findings demonstrate that contagious pig diseases in free-range systems are sustained through reinforcing feedback relationships that link poverty, husbandry practices, informal trade, and weak health and sanitation infrastructures.

The SGMB processes through FGD, helped identify the value chain actors involved in the Gwembe pig value chain, consistent with Glenn et al. [38] and Rich et al. [39]. It also revealed the unique composition of the value chain, including overlapping roles, where farmers may simultaneously act as traders or transporters. This reflects the informal and adaptive nature of rural livestock economies in many Low- and Middle-Income Countries (LMIC) settings. Through participatory mapping and discussion, stakeholders were able to collectively identify how geography, market access, seasonal livelihood dynamics, and pig management practices interact to shape disease vulnerability within the district.

A major contribution of the study lies in demonstrating how systems-thinking approaches can illuminate the non-biological determinants of livestock diseases. These included poverty, free-range husbandry, sanitation practices, informal pig trade, limited veterinary services, water scarcity, and low disease awareness. The SGMB process enabled participants to move beyond isolated explanations of disease occurrence and instead conceptualize disease transmission as an emergent property of interacting human, animal, and environmental systems. This finding aligns with Adam and de Savigny [24] who argue that systems thinking provides a more holistic understanding of health problems than conventional linear approaches. The findings further demonstrated that socio-economic vulnerability strongly influences husbandry practices and disease exposure pathways. Financial constraints were repeatedly identified by stakeholders as limiting the ability of farmers to invest in pig confinement, biosecurity infrastructure, veterinary care, and improved sanitation. These findings are consistent with previous studies demonstrating that poverty constrains disease prevention capacities among smallholder livestock farmers [9]. Within the ASF causal loop diagram, this relationship formed a reinforcing poverty–biosecurity loop, whereby low household income limited investment in biosecurity measures, increasing ASF risk and pig losses, which subsequently further reduced household income and resilience. This feedback structure illustrates how disease persistence may become self-reinforcing in resource-constrained livestock systems.

The predominance of free-range pig production in Gwembe emerged as another important systems driver influencing both ASF and PCC risks. Participants explained that free-range systems are economically attractive because they reduce feed and housing costs and allow pigs to scavenge for food and regulate body temperature naturally. However, stakeholders also linked these practices to increased exposure to contaminated environments, uncontrolled pig movement, and contact between infected and susceptible animals. Similar observations have been reported in other African smallholder pig systems where free-range husbandry has been associated with increased vulnerability to ASF and cysticercosis transmission [7,9,21]. Importantly, the findings suggest that free-range husbandry cannot be understood solely as a behavioural choice, but rather as an adaptive livelihood strategy shaped by poverty, environmental conditions, and limited access to production resources. The study also highlights PCC as a NTD situated at the intersection of livestock systems, sanitation, and human health inequities. Although stakeholders demonstrated some awareness of pig diseases generally, knowledge regarding PCC transmission pathways and associated human health risks was limited. Participants frequently associated pigs with environmental “cleaning” functions due to scavenging behavior, illustrating how locally normalized sanitation practices may inadvertently sustain transmission cycles. The PCC causal loop diagram (Fig 4B) demonstrated how low household income, poor sanitation infrastructure, open defecation, environmental contamination, and pig scavenging interact to reinforce disease persistence within the community.

Importantly, the human health implications of PCC extend beyond livestock productivity losses. PCC forms part of the transmission cycle of *Taenia solium*, a zoonotic parasite responsible for taeniasis and neurocysticercosis in humans. Neurocysticercosis is recognized as one of the leading preventable causes of epilepsy in endemic regions and disproportionately affects poor rural populations with inadequate sanitation and limited healthcare access. Consequently, the persistence of PCC within Gwembe reflects broader structural challenges involving water access, sanitation systems, public health awareness, and integrated disease surveillance. These findings reinforce the relevance of One Health approaches that recognize the interconnectedness of human, animal, and environmental health systems.

The systems perspective generated through SGMB also revealed how environmental and infrastructural conditions shape disease transmission pathways. Stakeholders described how seasonal water scarcity, poor sanitation, and the topography of Gwembe may facilitate contamination of shallow wells and agricultural environments through runoff from upland areas where open defecation occurs. Such observations suggest that disease exposure pathways are embedded within broader ecological and settlement dynamics rather than isolated husbandry practices alone. Similar socio-environmental relationships have been reported in studies examining cysticercosis transmission in endemic regions of sub-Saharan Africa [20–22,40]. The findings therefore support growing evidence that effective control of zoonotic neglected tropical diseases requires integrated interventions addressing both biomedical and structural determinants of transmission.

Trade dynamics emerged as another important system driver influencing disease spread. Stakeholders described extensive informal pig movement to Lusaka and Kasumbalesa, motivated primarily by market demand and income generation opportunities. However, discrepancies between stakeholder-reported transport volumes and official veterinary records suggested the existence of underreported or informal pig movements that may weaken disease surveillance systems. Rather than treating these inconsistencies as methodological weaknesses, triangulation across SGMB discussions, KIIs, and document review revealed them as analytically important indicators of porous market systems and potential surveillance gaps. Similar findings have been reported in studies linking informal livestock trade networks with transboundary disease spread in Africa [41]. Within the ASF CLD (Fig 4), informal pig movement formed a reinforcing trade loop where porous markets and weak movement monitoring potentially increased disease transmission risks beyond the geographical confines of Gwembe.

Importantly, the study suggests that control of ASF and PCC in free-range systems cannot rely solely on biomedical interventions or farmer behavior change campaigns. The reinforcing feedback loops identified through the CLDs indicate that disease persistence is embedded within interacting socio-economic and infrastructural constraints. For example, promoting pig confinement without addressing poverty and feed access may be unrealistic for resource-constrained households. Similarly, sanitation campaigns may have limited effectiveness if underlying barriers such as water scarcity, weak infrastructure, and low institutional support remain unaddressed. These findings therefore support integrated One Health approaches that simultaneously address livestock management, sanitation, community awareness, veterinary access, and livelihood vulnerability.

Several practical leverage points emerged from the systems analysis. These include strengthening community-based veterinary outreach, improving farmer education on ASF and PCC transmission pathways, promoting low-cost pig confinement approaches, strengthening meat inspection systems, improving WASH infrastructure, and integrating public health and veterinary surveillance activities. Community-based participatory approaches may be particularly important in Gwembe, where stakeholders and veterinary personnel described resistance toward externally introduced interventions and sanitation practices. By incorporating local knowledge systems and community priorities into intervention design, participatory approaches may improve both uptake and sustainability of disease-control strategies.

This study had several limitations. The SGMB session involved a relatively small number of purposively selected stakeholders, and findings therefore reflect context-specific perspectives rather than statistically representative patterns. Although thematic sufficiency was reached during the participatory discussions, conducting additional sessions across different regions of Gwembe and involving a broader range of stakeholders may have generated further insights into local system variation. Furthermore, the CLDs developed in this study represent qualitative conceptual models rather than empirically parameterized system dynamics simulations. Consequently, the relationships illustrated should be interpreted as stakeholder-perceived system structures and influence pathways rather than definitive causal mechanisms. Future research should therefore focus on quantitatively validating and parameterizing these relationships to support scenario modelling and intervention evaluation.

Nevertheless, the study demonstrates the utility of SGMB as a systems-based qualitative methodology for investigating complex livestock and neglected tropical disease systems. By integrating stakeholder perspectives, spatial mapping, temporal trends, institutional records, and systems modelling, the approach provided a richer understanding of the interacting drivers sustaining disease transmission within free-range pig systems. The findings contribute to growing evidence that sustainable control of zoonotic and livestock diseases in resource-constrained settings requires systems-oriented, participatory, and contextually grounded One Health strategies rather than isolated technical interventions alone.

## 5. Conclusion and policy recommendations

This study demonstrated that the transmission dynamics of ASF and PCC in free-range pig systems are shaped by interacting socio-economic, environmental, behavioural, trade-related and institutional determinants rather than biological factors alone. Through SGMB, stakeholders identified poverty, free-range husbandry practices, poor sanitation, informal pig trade, limited veterinary services, and low disease awareness as key system drivers influencing disease persistence and spread in Gwembe District.

The findings suggest that disease transmission is sustained through reinforcing feedback loops linking household poverty, inadequate biosecurity, environmental contamination, informal livestock movements, and weak access to animal and public health services. These interactions highlight the importance of adopting systems oriented and One Health approaches that address the underlying structural conditions that facilitate disease transmission.

Particularly for PCC, the study highlights the close interconnection between animal health, environmental sanitation, and human health. The persistence of PCC reflects broader challenges associated with inadequate sanitation infrastructure, limited awareness of *Taenia solium* transmission, and conditions that facilitate environmental contamination. Consequently, PCC should be viewed not only as a livestock disease but also as a NTD requiring coordinated interventions across veterinary, public health, and environmental sectors.

### 5.1. Policy implications of the study

The findings of this study highlight that the transmission and persistence of African swine fever (ASF) and porcine cysticercosis (PCC) in free-range pig systems are shaped by interconnected socio-economic, environmental, and institutional factors rather than biological processes alone. Consequently, effective disease control policies must adopt a systems-based and One Health approach that simultaneously addresses animal health, human behavior, environmental sanitation, and market dynamics. The study identified several leverage points that can strengthen disease prevention and improve the resilience of free-range pig production systems in Gwembe and similar settings.

A key policy priority is the strengthening of community-based veterinary services and participatory disease surveillance. Limited access to veterinary personnel was identified as a major constraint affecting disease awareness, reporting, and implementation of preventive measures. Expanding veterinary outreach programmes, improving community-level surveillance systems, and increasing engagement between veterinary officers and pig farmers would enhance early detection and reporting of disease outbreaks. Particular attention should be directed toward informal pig trade networks and transportation routes, where disease transmission risks may remain undetected by formal surveillance systems.

The study also demonstrates the need for policies that promote affordable and context-appropriate biosecurity measures. Financial constraints were identified as significant barriers preventing farmers from adopting improved husbandry practices. Rather than emphasizing costly confinement systems that may be unattainable for smallholder farmers, interventions should focus on low-cost biosecurity options such as simple pig housing structures, locally available fencing materials, improved waste management, and farmer-led biosecurity training. Access to microfinance, revolving livestock funds, and targeted livestock development programmes could further reduce financial barriers and facilitate investment in disease prevention.

The findings additionally underscore the importance of integrating water, sanitation, and hygiene (WASH) interventions into livestock health programmes. Poor sanitation, environmental contamination, and widespread open defecation were identified as important contributors to PCC transmission. Disease control efforts should therefore extend beyond livestock-focused interventions and incorporate community-based sanitation initiatives, improved access to safe water, and health education programmes that emphasize the human health consequences of Taenia solium infection, including neurocysticercosis. Integrating veterinary and public health sectors would strengthen efforts to address the shared human-animal-environment interface that underpins disease transmission.

Finally, the study highlights the value of participatory approaches in policy design and implementation. The Spatial Group Model Building process demonstrated that local stakeholders possess valuable contextual knowledge regarding disease drivers, market dynamics, and feasible intervention strategies. Policies developed through participatory engagement are more likely to be accepted, adopted, and sustained because they reflect local realities and foster community ownership. Future disease control strategies should therefore incorporate stakeholder participation throughout planning, implementation, and evaluation processes to ensure interventions are both effective and socially acceptable.

Taken together, these findings suggest that sustainable control of ASF and PCC in free-range pig systems requires integrated policies that combine strengthened veterinary services, affordable biosecurity measures, improved sanitation, and participatory governance within a comprehensive One Health framework. Such approaches have the potential not only to reduce disease risks but also to improve rural livelihoods, food security, and public health outcomes.
